# Role of the Ribonuclease ONCONASE in miRNA Biogenesis and tRNA Processing: Focus on Cancer and Viral Infections

**DOI:** 10.3390/ijms23126556

**Published:** 2022-06-12

**Authors:** Marta Menegazzi, Giovanni Gotte

**Affiliations:** Biochemistry Section, Department of Neuroscience, Biomedicine and Movement Sciences, School of Medicine, University of Verona, Strada Le Grazie, 8, I-37134 Verona, Italy; giovanni.gotte@univr.it

**Keywords:** onconase, microRNA, tRNA fragments, viral infection, proteins expression, cancer, RNA interfering, COVID-19

## Abstract

The majority of transcribed RNAs do not codify for proteins, nevertheless they display crucial regulatory functions by affecting the cellular protein expression profile. MicroRNAs (miRNAs) and transfer RNA-derived small RNAs (tsRNAs) are effectors of interfering mechanisms, so that their biogenesis is a tightly regulated process. Onconase (ONC) is an amphibian ribonuclease known for cytotoxicity against tumors and antiviral activity. Additionally, ONC administration in patients resulted in clinical effectiveness and in a well-tolerated feature, at least for lung carcinoma and malignant mesothelioma. Moreover, the ONC therapeutic effects are actually potentiated by cotreatment with many conventional antitumor drugs. This review not only aims to describe the ONC activity occurring either in different tumors or in viral infections but also to analyze the molecular mechanisms underlying ONC pleiotropic and cellular-specific effects. In cancer, data suggest that ONC affects malignant phenotypes by generating tRNA fragments and miRNAs able to downregulate oncogenes expression and upregulate tumor-suppressor proteins. In cells infected by viruses, ONC hampers viral spread by digesting the primer tRNAs necessary for viral DNA replication. In this scenario, new therapeutic tools might be developed by exploiting the action of ONC-elicited RNA derivatives.

## 1. Introduction

Many drugs used in disease therapy are designed to target proteins. However, hurdles can arise when their structure is totally unknown or their hydrophilic flat surface makes the interaction with the drug difficult [1,2]. On the contrary, the modulation of mRNAs availability can be an alternative and attractive therapeutic strategy [3]. Importantly, because RNAs are upstream of proteins, this approach may allow to affect proteins as well [1].

Although 90% of human DNA is transcribed into RNA, only 2% of the genome encodes for known proteins [1,4]. Hence, most of the transcribed RNA should hold up non-protein-coding functions. Non-coding RNAs (ncRNAs) have been subsequently found to display many different and complex functions [4]. They play a key role in carcinogenesis and tumor progression, or in viral infection as well, for their ability to control mRNAs stability and translation [1,5]. Remarkably, gene and protein expression are affected by the presence in the cell of several ncRNA species, including microRNAs (miRNAs), long non-coding RNAs (lncRNAs), circular RNAs (circRNAs), piwi-interacting RNAs (piRNAs) and transfer RNA-derived small RNAs (tsRNAs), which can also reciprocally interact. Therefore, the final effect exerted on the cell phenotype derives from the whole ncRNAs expression profile and results from many activating and/or inhibiting molecules targeting these RNAs [6].

We aim here to analyze the plausible mechanisms by which ribonucleolytic cleavages of ncRNA precursors can control the pathogenic cell phenotype, focusing our attention on miRNA and tsRNA biogenesis in cancer and viral infection. The data so far available match very well with the effects exerted by onconase, an amphibian ribonuclease.

## 2. Micro RNA Biogenesis and Function

miRNAs are small, about 20–24 nucleotides ncRNA species, which control mRNAs degradation and/or translational repression of their target genes. Copies of a single miRNA can simultaneously bind several targets, thus inhibiting the expression of various proteins, whereas different miRNAs can in turn target a single mRNA. Therefore, pleiotropic cellular functions can be achieved by tuning miRNAs extent [7]. Accordingly, a dysregulation of miRNAs expression is associated with human diseases and cancer [8].

**Figure 1:**
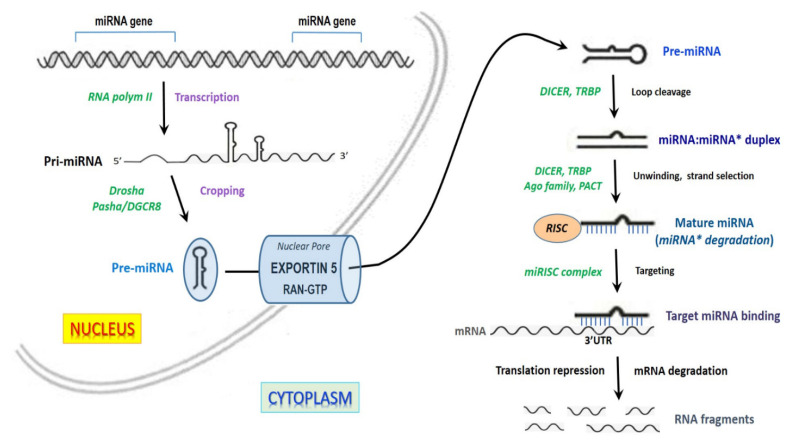
Schematic representation of microRNA (miRNA) biogenesis. In the nucleus, the first event is the miRNA transcription by RNA polymerase II (Pol II). Most genes encoding miRNAs are located in introns and contain their own promoter regions. Following the transcription of long primary transcripts, Drosha, a type III RNase, along with the cofactor Pasha/DGCR8 protein, binds to and cleaves the 3′ and 5′ strands of the primary miRNA (pri-miRNA) transcript, hence generating the pre-miRNA. Next, the Exportin 5+RAN-GTP complex mediates the shift of pre-miRNAs from the nucleus into the cytoplasm, where the RNase III Dicer and the TAR RNA binding protein (TRBP) cleave the pre-miRNA terminal loop, resulting in a miRNA:miRNA* duplex. The duplex is processed by the argonaute (AGO) proteins family that act in concert with cofactors, such as PACT. This induces the unwinding and the strand selection that drives to miRNA* strand degradation and mature miRNA production. Mature miRNA is in turn incorporated into the RNA-induced silencing complex (RISC) and driven to target mRNAs with complementary sites, resulting in translational repression or mRNA degradation.

miRNAs are transcribed as longer miRNA precursors: primary miRNAs (pri-miRNAs) are processed first to precursor miRNAs (pre-miRNAs) then to mature miRNAs by two endonucleolytic cleavages driven by multiprotein complexes, including the RNase III enzymes Drosha in the nucleus and Dicer in the cytoplasm [7,8] (Figure 1). Since miRNAs play a key role as protein expression controllers, their biogenesis is finely regulated in response to change in the cellular conditions [7]. For instance, on one hand the tumor suppressor p53 interacts with the Drosha complex in cancer cells, in this way facilitating the maturation of a restricted population of pri-miRNAs in response to DNA damage [7,9]. On the other hand, the lin28 homolog A protein can block the processing of pri-let-7 and pre-let-7, or of other pri- and pre-miRNAs by inhibiting the association of pri-let-7 and pre-let-7 with Drosha/Dicer complexes [10]. Again, the KH-type splicing regulatory protein (KSRP) is a component of both Drosha and Dicer complexes and regulates the biogenesis of a subset of miRNAs by recognizing specific sequences, such as two GGG triplets [11].

In summary, as it occurs for the DNA-binding transcription factors, the regulation of miRNAs maturation needs the coordinated action of coactivator and corepressor RNA-binding proteins [7].

To reach the inhibitory effect on mRNA translation, mature miRNAs generally bind to their mRNA targets in the mRNA’s 3′untranslated region (UTR) site, although noncanonical binding sites also exist [12]. However, the cellular miRNA effects depend on the functions of the target genes they repress. In cancer, miRNAs targeting tumor-suppressor genes are usually upregulated, whereas miRNAs that target oncogenes are downregulated. In this way, the oncogene functions are preserved while the ones of the suppressor gene are deleted. Additionally, a complex crosstalk between miRNAs and signal transduction occurs, since both transcription factors and miRNA biogenesis are controlled in a bi-univocal manner by cell signaling [8]. Indeed, many signaling pathways regulate upstream the miRNAs processing machinery, as well as miRNAs expression controls cell signaling downstream [8], so that it is often difficult to discern the actual cause or effect of such a functional event.

## 3. tRNA-Derived Small Interfering RNAs

Transfer RNAs (tRNAs) are 73–90 nucleotides-long polymers displaying characteristic secondary and tertiary structures. tRNAs are structured by base-paired stems interchanged by unpaired regions called D-loop, anticodon-loop, variable-loop and TψC-loop [13]. In addition to a canonical role played in the protein synthesis machinery [14], tRNAs and their cleavage products can act as signaling molecules under stress, and/or as gene expression regulators [14,15]. Moreover, as discussed later, they can also be primers for viral replications [13,16]. Therefore, it is not surprising that tRNAs are so abundant in the cell, since they represent about 15% of total RNA species [15]. Indeed, a high redundancy of about 500 tRNA genes present in the human genome decode only 61 different anticodons, although many of these genes are poorly expressed [17]. Torres et al. claimed that tRNAs displaying the same anticodon sequence are functionally equal in terms of genetic translation, while their differential expression could be related to noncanonical functions [17].

**Figure 2:**
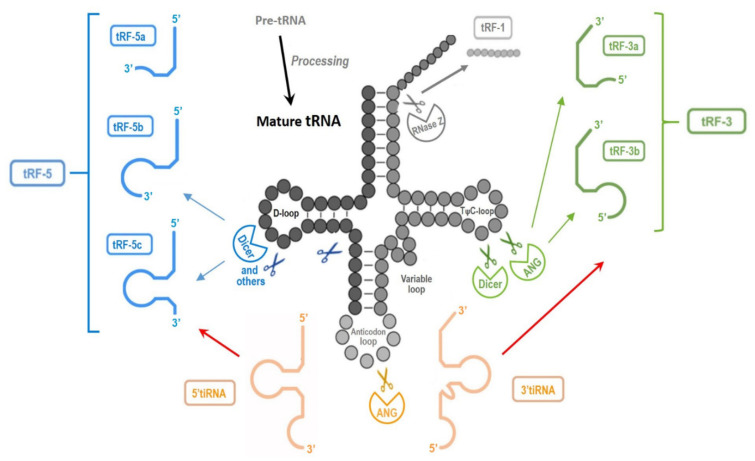
tRNA degradation producing different tRNA-derived fragments, either from pre-tRNA or from mature tRNA. During tRNA processing, RNases remove the pre-tRNA transcripts. tRF-1s (gray) are produced by the RNase Z cleavage of pre-tRNA. Then, mature tRNA contains multiple modified nucleosides and can be cleaved in the anticodon loop by angiogenin (ANG) to produce tiRNA-5 and tiRNA-3 series (orange). Then, a tRF-5 series is produced by the RNase III Dicer, and/or by other actors, from the 50- ends of mature tRNAs (cyan): these enzymes cleave the substrate either at the D-loop region or in sites located between it and the anticodon-loop. Instead, a cleavage in the TψC-loop operated by Dicer or by ANG results in the production of the tRF-3 series (green). Both tRF-5 and tRF-3 may also be processed from the tiRNA-5 and tiRNA-3 series (red arrows).

tRNAs can be cleaved to generate a heterogeneous class of tRNA fragments [18] (Figure 2). Among them, tRNA-derived stress-induced RNAs (tiRNAs) result from the cleavage occurring at the anticodon-loop. The tiRNA products are about 31–40 nt-long derivatives, differentiated in 5′tiRNA or 3′tiRNA, as a function of the availability of the 3′ or 5′ end at the anticodon cleavage site [18]. Angiogenin (ANG), a ribonuclease (RNase 5) [19,20] secreted by stressed cells [21], can generate tiRNA products as a result of paracrine signaling [22]. Since ANG normally elicits prosurvival signaling, many resulting tiRNAs facilitate the cellular response to stress by reprogramming translation, hence inhibiting apoptosis and degrading mRNAs [22]. Recent studies report that tiRNAs are significantly involved in cancer development: indeed, the sex hormone signaling pathway promotes ANG-mediated tRNA cleavage, generating tiRNA species called sex hormone-dependent tRNA-derived RNAs (SHOT-RNAs). 5′-SHOT-RNA^AspGUC^, 5′-SHOT-RNA^HisGUG^, as well as 5′-SHOT-RNA^LysCUU^, but not their 3′-SHOT-RNA counterparts, are required for the proliferation of prostate cancer cells [23]. The same 5′-SHOT-RNAs showed a prominent expression level in human breast cancer specimens, in comparison with normal breast tissues [23,24]. Although tiRNAs principally induce cell survival signaling, Mo et al. found that 5′-tiRNA^Val^ acts as a tumor suppressor in breast cancer through a mechanism involving the Wnt/β-Catenin signaling pathway [25].

In addition to tiRNA products, other even shorter fragments called tRNA-derived small RNA fragments (tRFs), that seem preferentially involved in gene expression regulation, can result from tRNAs cleavage [18]. tRFs are distinguished in at least three types, called tRF-5, tRF-3 and tRF-1 [26]: tRF-5 and tRF-3 derived, respectively, from the 5′-end of tRNA upon the D-loop cleavage, and from the 3′-end upon the TψC-loop cleavage [26]. tRF-1 are instead generated from the cleavage of tRNA precursors at the 3′ end [18]. Additionally, a tRF-5 *a*, *b* or *c*, and of tRF-3 *a* or *b* subclassification has been advanced, in which a different length characterizes each subclass, thus demonstrating a high heterogeneity of the resulting tRFs [27] (Figure 2). Importantly, CLASH (cross-linking ligation and sequencing of hybrids) data suggest that the majority of tRF-3s and some tRF-5s can interact with complementary sequences of mRNA targets to regulate their expression or functionality [28].

The mechanism responsible for the production of each tsRNA type is challenging. As mentioned, under stress conditions, ANG cleaves tRNAs at the anticodon-loop, producing 5′tiRNAs and 3′tiRNAs [22], although it was reported that ANG cleaves only a subset of tRNAs. By contrast, the other tiRNAs are ANG-independent [29], since tiRNAs have been found in ANG knockout cells [30]. Therefore, other unknown RNases may be involved in the production of tiRNA species. As for tRFs, some studies showed the generation of tRF-3s and tRF-5s is related to multiple RNase members, including ANG and Dicer. However, the role of Dicer in tRFs generation is still debated, since tRFs are detectable also in Dicer knockout mouse fibroblasts [31]. Again, the analysis of tRFs deriving from primary tRNA trailer sequences shows the 5′end of tRF-1 matches with the RNase Z cleavage site [28,32], whereas the biogenesis of tRF-1001 is catalyzed by the ELAC2 endonuclease that cleaves pre-tRNAs [33].

Although the tRFs biogenesis is distinct from that of miRNA, their properties are similar. Indeed, in diseases, cancer in particular, they play a major role in RNA silencing [26,28]. For instance, some specific tRFs derived by cleaving the tRNAs coding for Glu, Asp and Gly can suppress the stability of multiple oncogenic transcripts in breast cancer cells by displacing their 3′ UTRs from the protumor RNA-binding protein YBX1 [34]. Again, tRF-3027 suppresses cell proliferation and modulates DNA-damage response [35]. On the contrary, tRF-Lys-010 promotes triple-negative breast cancer (TNBC) proliferation and migration, and is significantly increased in human specimens from TNBC, hence indicating an emerging oncogenic action [36].

In brief, tRFs are not random tRNAs degradation products, highly conserved in all domains of life [28]. Nevertheless, the mechanism of tRNAs processing as well as the role of the involved RNases effectors should require further investigations. Anyway, many studies focused on tRFs functions recently received increasing attention, so that a more complete information and literature revisions are available [37,38,39,40].

## 4. RNases and RNA Processing as Tumor and Viral Infection Management

### 4.1. RNases: A Vast Family Devoted to RNA Control

Being that RNA processing is directly linked to protein synthesis and, therefore, to cell life cycle, the activity of RNases is crucial. Although being too numerous to be discussed in a single review, the RNases most involved in antitumor or antiviral studies are mentioned hereafter.

Many reviews described the properties of the secretory “pancreatic-type” (pt), or RNase A-like, RNases [20,41,42,43,44,45], so called because they display structural and functional similarities with the well-known bovine pancreatic RNase A [46]. Among the most known present in humans, and numbered as RNase 1–8, human pancreatic (HP)-RNase (RNase 1), angiogenin (RNase 5) are noteworthy. Beyond humans, mammalian bovine seminal (BS)-RNase intrinsically displays a relevant antitumor activity in mice and human cells [47,48]. Then, some RNase 1 and 5 mutants able to escape the cellular RNase inhibitor (RI) [49] are remarkably cytotoxic [50,51]. Again, as we reported in the previous chapter, RNase 5 plays a crucial role in tRNA cleavage.

Moreover, RNase 2 and 3, being, respectively, the eosinophil-derived neurotoxin (EDN) and the eosinophil cationic protein (ECP), are involved in host defense [52,53], as well as fungal RNases, such as α-sarcin [54,55].

Then, while RNase H from hepatitis B or HIV viruses was only recently successfully targeted by drugs [56,57], human RNase P can cleave the RNA of hepatitis C virus [58] and, together with RNase Z, affects protein translation by processing the tRNA-like noncoding mascRNA [59].

Importantly, the cellularly ubiquitous RNase L targets, upon its activation, viral ssRNAs and protects human cells from infections [60].

Again, as mentioned above, the RNase III-like nuclear Drosha or cytoplasmic Dicer process either tRNAs or mRNAs.

Interestingly, the RNases of the T2 family, present in bacteria, plants, viruses and also in animals [61], have their human version, called RNASET2, that is secreted by damaged tissues to initiate immune response [62].

Importantly, other bacterial RNases display relevant anticancer activity and deserve to be mentioned. Belonging to the N1/T1 microbial superfamily, the most cytotoxic are Barnase from *Bacillus amyloliquefaciens* and above all Binase from *B. pimulus intermedius* [63,64,65]. More recently, Balifase from *B. licheniformis* and Balnase from *B. altitudinis*, also displayed to be cytotoxic, probably thanks to their supramolecular organization [66].

Finally, special attention is devoted in this review to amphibian RNases: many of them are included in the pt-RNase superfamily because of their structural and functional similarity to RNase A, and they became increasingly important for their antitumor and antiviral activities.

The RNases recovered from the liver of bullfrog *Rana Catesbeiana*, rc-RNases [67] or from the eggs of Japanese frog *Rana japonica* [68] were overpassed in importance by RNases derived from the Leopard Frog *Rana Pipiens* oocytes, i.e., Amphinase (Amph) and Ranpirnase, that was then called onconase (ONC). The sequence identity of the two latter enzymes is only about 40%; however, they are both basic, and their enzymatic activity mechanisms are qualitatively similar to RNase A, although they are definitely less enzymatically active than the proto-type [69].

Amph is 114 AA residues long and highly polymorphic, being split in four isoforms characterized by highly similar sequences but differently glycosylated profiles [70]. Although being remarkably cytotoxic [69], this heterogeneity somehow limited its usage in many functional applications.

Moreover, ONC is polymorphic, but only because of Ser/Thr polymorphism present in the 25 position [71]. However, this does not affect ONC enzymatic activity [69]. Another natural triple I11V/D20N/S103R ONC variant was found in oocytes, although encoded by another gene [72]. Anyhow, the high prevalence of the first isoform discovered in 1991 permitted ONC to be extensively used in many pathological contests.

### 4.2. The Amphibian Onconase (ONC): A RNase for Cytotoxicity

The peculiar nature of ONC [71] has acquired importance since the end of the last century. It is the smallest pt-RNase, being only 104 AA residues long and 11.8 kDa highly basic enzyme (pI 9.7) [69]. Beyond oocytes where is localized in the yolk, like rc-RNases [73], ONC is extracted also from *Rana Pipiens* early embryos. Indeed, although displaying only 30% sequence identity with RNase A [21], ONC displays the typical “V-like”, or “kidneylike” folding of pt-RNases, with a central cavity accommodating the RNA substrates [74], permitting it to be included in the pt-RNases superfamily [41]. ONC conserves a catalytic triad formed by one Lys and two His residues (H10/K31/H97), as well as the CKxxNTF consensus sequence including the active site Lys, a feature shared with all mammalian pt-RNases [71]. Then, the majority of ONC basic residues are located nearby the active site cleft, again like all pt-RNases [41].

It has been advanced that ONC is synthesized in the female frog liver to be then addressed through the bloodstream to oocytes during their maturation [73]. Therefore, its physiological role, although not extensively analyzed, seems devoted to defend oocytes themselves and early embryos from infections [75]. However, many frog RNases are carbohydrate-binding enzymes [68,76], assigning them a double, RNase plus lectin, function [77]. This would suggest they are also leczymes that are known to display antitumor activity [78], not in contrast with the ability of ONC to bind sialic-acid molecules present on the surface of malignant cells in humans and to induce their deathful agglutination [79]. Indeed, although the physiological role of ONC in amphibians is not still precisely defined, its application in mammals, in humans in particular, has been extensively investigated.

Importantly, ONC exerts many important biological properties in the host cells thanks to its ribonucleolytic activity: for example, Ardelt et al. [80] proposed that it could decrease the formation of ROS species, or even affect the mitochondrial transmembrane potential as well. Therefore, besides an important antiviral activity [81], ONC, thanks above all to its high stability and basicity, can exert remarkable cytostatic and cytotoxic actions [82] against many cancer types, as is extensively described later.

### 4.3. Determinants for the Antitumor Activity of ONC

ONC is able to counteract many still poorly curable tumors. Its advantage with respect to other potential drugs is its selectivity for malignant cells, favorited with respect to normal ones [69,83]. Conversely, since ONC is a secretory RNase, an obstacle for being actually cytotoxicity is its cellular internalization. The possibility for ONC to exploit a receptor [84] or, conversely, to be internalized in cells through endocytosis [85,86,87] is debated. In any case, data suggest that ONC can cross the cell membrane, although differently as a function of the cell type. RNases endocytosis requires a fruitful interaction with the membrane, for which both electrostatic or specific interactions are crucial [88]. Hence, the high basicity of ONC can justify its preferential action against tumor cells, since the membrane of malignant cells is richer in negatively charged sialic acid molecules [89] than the one of normal cells [90,91]. Once inside cells, another barrier for RNases is the cellular negatively charged RNase Inhibitor (RI), present either in the cytosol or in the nucleus [49,92]. RI is a 50 kDa horseshoe-shaped molecule forming very tight complexes with many natively monomeric mammalian RNases, such as RNase A, RNase 1 and ANG, with Kd values comprised between the pico- and the femto-molar range [93]. Instead, amphibian ONC can evade RI because it lacks many flexible regions and loops containing the key residues responsible for the RNase-RI complex formation [84,94]. The ONC–RI complex Kd, measurable only with salt concentrations lower than the physiological ones, is definitely higher than the ones of other pt-RNases [95]. Therefore, ONC can actually exert, for this reason above others, its remarkable intracellular biological activity.

### 4.4. ONC Antitumor Efficacy

Since its first discovery and purification from frog oocytes in the late 1980s in the past century, ONC has displayed to exert in vitro antitumor activity on human leukemic, submaxillary carcinoma and colon adenocarcinoma cell lines [96]. A preferential cytostatic effect was firstly registered, although without recognizing the intrinsic mechanism of ONC activity [96].

Later, ONC antitumor effects were also tested in vivo, such as in mice either bearing M109 Madison carcinoma [97] or colon carcinoma [98], and again in tumor xenografts of nude mice of A549 human lung cancer [99] or of ASPC-1 human pancreatic cancer [100]. ONC treatment resulted in dose-dependent (from 50 to 200 µg/mouse) tumor growth inhibition, as well as survival improvement.

Thereafter, other cell lines derived from squamous head–neck carcinoma [101], lymphoma [102], or from ovarian cancer [103] were affected by ONC.

Comparative studies have been carried out between ONC administered alone or in association with other treatments, such as tamoxifen, lovastatin, cisplatin [100,104], vincristine [98,105], rituximab, mafosfamide, doxorubicin, dexamethasone [105], cepharanthine [106], dihydroartemisinin [107], gemcitabine, everolimus [108], PARP inhibitors [83], dabrafenib [109], tumor necrosis factor-α [110], interferons [111,112], retinoic acid [113], rosiglitazone [114], ionizing radiation [115,116] and mild hyperthermia [117]. In vitro and in vivo data suggest the combination of ONC with such treatments that individually express lower cytotoxic profiles can result in a stronger antitumor potential, although the molecular mechanisms underlying these additive effects have been poorly investigated. Importantly, ONC was showed to also remarkably affect the viability of tumor cells after they acquire resistance to conventional chemotherapeutics [98,109,118].

The high efficacy either found in vitro or in preclinical studies allowed to experiment with ONC administration in patients. Since ONC targets RNA, it fruitfully does not display mutagenic activity in comparison with DNA-damaging antitumor drugs.

The first phase I clinical trial was carried out in 1993 in a small number of patients affected by a variety of relapsing and resistant solid tumors [119]. A weekly intravenous bolus ONC was injected in patients, with doses comprised between 60 mg/m^2^ and 960 mg/m^2^ [119]. Considering the advanced tumor state of patients, the results were promising because ONC was well-tolerated and exerted a broad spectrum of anticancer activity, demonstrating a stabilization of previously progressive diseases in 9.3% of patients [119]. Another phase I clinical trial started in 1995 by administering ONC in thirty patients affected by advanced nonsmall cell lung cancer [120]. ONC treatment resulted in the disease stabilization for 20% of patients with an overall median survival time of 7.7 months [120]. The phase I studies determined a maximum tolerated dose of 960 µg/m^2^/week, although the recommended dose for the successive phase II was halved to 480 µg/m^2^/week [121,122]. These first studies showed, however, that the dose-limiting toxicity for ONC was related to an albeit reversible nephrotoxicity [123], while other side effects included flushing and myalgia [119,120]. Instead, ONC did not induce the side effects associated with most antitumor drugs, i.e., myelosuppression, mucositis, alopecia, cardiotoxicity, coagulopathy, hepatotoxicity and adverse metabolic effects [121,122]. In brief, ONC displayed a favorable toxicity profile in comparison with other chemotherapy regimens [119,120,121,122].

The largest phase II trial was carried out with patients affected by malignant unresectable mesothelioma, 51% of which showed clinical effectiveness and an overall median survival of six months [122,123,124]. Conversely, dissatisfying results were obtained in the phase II trial vs. advanced breast cancer (2 clinical activities out of 17) [122], although the administered ONC dosage was lower (240 µg/m^2^/week) than the recommended one. Additionally, minimal activity was also registered in 14 patients bearing kidney cancer [121].

A phase III clinical trial was then performed with 154 patients harboring malignant mesothelioma [122,125,126]. Patients received either ONC (480 µg/m^2^/week) or doxorubicin (60 mg/m^2^ every three weeks for six cycles): a mild advantage of 11.6 vs. 9.6 months emerged in the average survival of ONC-treated patients vs. the doxorubicin-treated ones, respectively [125,126].

From all this evidence, ONC clinical trials were revealed to be limited, principally because ONC is a small protein formed by only 104 AA residues [71] that is therefore addressed to glomerular filtration and renal accumulation [123]. With the aim to enlarge the ONC moiety dimensions and reduce this side effects, the group lead by RT. Raines produced a Cys mutant, forming a covalently stabilized cyclic trimer upon reacting with a trifunctional maleimide, although this derivative was less cytotoxic than the wt-ONC monomer [127]. Again, in order to enlarge the ONC moiety dimensions, as well as to increase the selectivity of the druglike derivatives for malignant cells, ONC was derivatized with specific human antibodies. In this way, an ONC–CD22 monoclonal antibody conjugate was active vs. non-Hodgkin lymphoma [128]. Then, ONC fusion with human serum albumin provided activity either against HT29 colorectal carcinoma or A375 melanoma cell lines. Importantly, cytotoxicity was registered in mice as well, together with a reduced renal accumulation [129]. Moreover, toxicity against head–neck EGFR-expressing tumor cells was registered upon conjugating ONC with a dengue virus-derived peptide [130]. Moreover, ONC fusion with the transferrin N-terminal domain gained activity against HepG2 and Hela cells [131], while fusion with the scorpion-derived chlorotoxin made ONC potently active against U251 and SHG-44 glioma cells [132]. A similar approach was also applied with diabodies, i.e., noncovalent dimers of ONC conjugated with dimeric antibody fragments (scFv) derived from variable regions of their heavy and light chains [45]. In particular, ONC dimeric derivatives were produced with an anti CD22 scFv diabody [133] with humanized anti-Trop-2 [134], or with anti-EGFR as well [135]. These adducts were remarkably cytotoxic, and the latter two were also active in mice with no dose limiting [134,135].

Interestingly, we recently found that wt-ONC can dimerize through the swapping [136] of its N-terminal ends [137]. In this way, the dimer reconstitutes the active site geometry and maintains satisfactory catalytic and antitumor activity levels *vs.* human pancreatic [138] and melanoma cell lines [137]. Although requiring further tuning, this approach might represent a promising perspective considering that the effects registered in vivo suggested to increase ONC moieties dimensions with respect to the native monomer. Indeed, the enlargement of its dimensions would probably help ONC to avoid renal uptake and contemporarily increase the ONC half-life circulating in the bloodstream.

### 4.5. Cytostatic and Cytotoxic Activity of ONC in Cancer: Mechanism Involving Gene and/or Protein Expression Profile Alteration

The ONC targets and the effects registered in the studies mentioned hereafter are summarized in Table 1. The first investigation carried out on cultures of different tumor cell types highlighted that the ONC antitumor effectiveness is time- and concentration-dependent [96]. ONC required from one to two days latency to slow down cell proliferation, to arrest the cell cycle in the G_1_ phase and to reduce cell number in S phase, while at longer times a concentration-dependent cell death was registered [96]. Juan et al. confirmed that a perturbation of the cell cycle progression, leading to the accumulation of cells in the G0/G1 phase, occurred in ONC-treated U936 lymphoma cells. Additionally, a dysregulation of protein expression involved in the cell cycle progression took place [139]. Finally, a cyclin D3 downregulation, an upregulation of p16^INK4A^ and p21^WAF1/CIP1^ and an induction of p27^KIP1^ were registered in ONC-treated U936 cells [139].

The Rybak’s group reported the Ras-transformed NIH/3T3 active cycling cells were more sensitive to ONC cytotoxicity, as compared with the quiescent wt-NIH/3T3 ones [140]. A decreased number of cells in the S phase was reported concurrently with an increase in apoptosis, hence suggesting that blocking cells in the S phase could make them irreversibly leave the cell cycle to enter the cell death pathway [140].

Iordanov et al. showed that ONC induces HeLa cells’ death in a caspase-dependent but P53-independent manner and without massive cytochrome c release or BAX translocation from the cytosol to mitochondria [141]. In another paper, the same authors reported an early activation of c-Jun NH_2_-terminal kinase (JNK) and a p38 mitogen-activated protein kinase (MAPK) in HeLa cells and mouse embryo fibroblasts exposed to proapoptotic ONC concentrations [142]. Then, they assessed that JNKs are important mediators of ONC-elicited cell death using fibroblasts characterized by targeted disruption of both JNK1 and JNK2 alleles. However, the knockdown of these kinases did not completely abrogate the ONC-induced apoptosis [142].

**Table 1:** Effects of ONC on tumors and/or in tumor cells.

Tumor Type (H = Human)	Cell Type	General Biological Effect(s)	Intracellular Targets	Reference
H Lymphoma	U937	G1/S arrest, cytostatic effect	P16INK4A ↑ P21WAF1/CIP1 ↑ P27KIP ↑ Rb phosphoryl ↓ Cyclin D3 phosphoryl ↓	Juan, G. [139]
Mouse embryos	NIH/3T3 Sarcoma/lymphoma sensitive fibroblasts	Cell cycle braking	-	Smith, M.R. [140]
H Cervix carcinoma	HeLa tk-	t-RNA targeting Apoptosis ↑ p53 independent	Cleaved caspases 9, 3, 7 ↑	Iordanov, M.S. [141]
H Cervix carcinoma	HeLa tk-	Apoptosis ↑	SAPK1 (JNK1 and JNK2) ↑ SAPK2 (p38 MAP-K) ↑ I-κB ↔ NF-κB	Iordanov, M.S. [142]
H Malignant Pleural Mesothelioma	MPM H2595, H2373 and H2591	cell proliferation ↓ invasion ↓ miRNAs ↑↓	NF-κB1 ↓ hsa-miR-17* ↓ hsa-miR-30 ↑	Goparaju, C.M. [143]
H Malignant Mesothelioma	REN (epithelioid) PPM-Mi (sarcomatoid)	Tumor mass in mice ↓	NF-κB nuclear traslocation ↓ MMP9 secretion and activity ↓	Nasu, M. [144]
Leukemia	Jurkat T-lymphocytic Jurkat-BαM	cell proliferation ↓ (72/96h)	NF-κB ↓	Tsai, S.Y. [145]
H Breast carcinoma Leukemia	T47D (duct breast) HL-60 Jurkat-SN, Jurkat-BαM	mitochondrial transmembrane potential ↓, ATP ↓	Bcl-2 ↓, Bax ↑ Catalase ↑ (Jurkat cells)	Ardelt, B. [80]
H Malignant Mesothelioma	M25, M29, M35, M42, M49	Cell proliferation ↓ t-RNA damaging	ATF3, IL24, IL6, COX-2, PTOV1 modulation (cell line dependent)	Altomare, D.A. [146]
H and murine Leukemia H colon adenocarcinoma	HL-60, A-253, Colo 320	G1/S cell cycle arrest RNA content ↓ Colony population and size ↓ Proliferation ↓	-	Darzynkiewicz, Z. [96]
H neuroblastoma	UKF-NB-3, IMR-32	G1 cell cycle arrest Caspase-indep. cell death	-	Michaelis, M. [118]
H breast carcinoma H lung carcinoma	T47D, MCF7, MDA-MB-231, H292	ONC + rosiglitazone synerg. cytotoxicity ↑ Apoptosis ↑	PI3K ↓, Fra-1 ↓ Survivin ↓	Ramos-Nino, M.E. [114]
H pancreatic adenocarcinoma	Panc1, PaCa44	Cell proliferation ↓ ROS-dependent Akt/mTOR autophagic cell death ↑	Beclin1 ↑ LC3-II ↑ UCP2 ↓ MnSOD ↓	Fiorini, C. [108]
H malignant melanoma	A375	Cell proliferation ↓ PARP inhibitors sensitiz. ↑	γ-H2AX ↑ (with AZD) NF-κB ↓, TNF-α ↓, Cleaved PARP ↑	Raineri, A. [83]
H malignant melanoma	Parental A375; Dabrafenib resistant A375DR	Cell proliferation, migration, invasion ↓ Colony Formation ↓	p65 NF-κB ↓ IKK phosphoryl ↓ MMP2 ↓	Raineri, A. [109]
H malignant melanoma	A375, MeWo (ONC monomer and dimer)	Cell proliferation ↓ Colony Formation ↓ Apoptosis ↑	MMP2 ↓ STAT3 ↓ pTyrSTAT3 ↓ pSerSTAT3 ↓, pSrc ↓	Gotte, G. [137]
H malignant melanoma	A375, FO1	Cell viability ↓	miR-20a-3p ↑, miR-29a-3p ↑ miR-34a-5p ↑ Cyclin D1 ↓, Cyclin A2 ↓ P21 ↓, P27 ↓ ERK ↓, HIF1_α ↓, PDK1 ↓, CREB ↓, SIRT1 ↓, SOX2 ↓, Fra1↓, AXL ↓, cMet ↓, AKT ↓, ZO1 ↓, uPAR ↓	De Tomi, E. [147]

Nuclear factor-kappa B (NF-κB) is a transcription factor functionally associated with cell survival since it is involved in a multitude of critical cellular functions, including cell proliferation and apoptosis [148]. Notably, NF-κB inhibition decreases the cell malignancy potential and increases the animal lifetime in mesothelioma cells and mesothelioma mouse xenografts, respectively [149]. In mesothelioma cells, ONC downregulates the expression of NF-κB and of its target genes, such as the ABC transporter, the apoptosis regulator Bcl extra-large (Bcl-xl), the inhibitor of apoptosis (cIAP) and the metalloproteinase-9 [143,144]. Again, a significant suppression of cell proliferation took place with a concomitant apoptosis induction after incubating ONC in Jurkat leukemia cells, in which antitumor effects were closely coordinated with NF-κB downregulation [145]. Notably, NF-κB also exerts pleiotropic proliferative effects in malignant melanoma [150], and we recently reported that ONC elicited the inhibition of NF-κB DNA-binding through the downregulation of its target gene tumor necrosis factor (TNF)α in the human A375 melanoma cell line [83]. In addition, an increased apoptotic cell death was registered in cells treated with a poly (ADP-ribose) polymerase (PARP)-1 inhibitor before administering ONC. In this way, a pretreatment with a drug able to block DNA repair, as it is a PARP-1 inhibitor, strongly presensitized A375 cells to suffer a subsequent cytotoxic effect triggered by ONC [83]. In another paper, we compared the ONC effectiveness in A375 parental cells with an A375 cells’ subpopulation that had become resistant to dabrafenib, a well-known BRAF-inhibitor targeting BRAF-mutated melanoma [109]: ONC reduced the nuclear NF-κB expression level, the activation of its upstream kinases (IKKs), as well as the activity of its target metalloproteinase-2, irrespectively of the subpopulations tested. Remarkably, ONC was also able to brake cell colony formation, as well as migration and invasion capability in both cell subpopulations [109].

Again, Michaelis and coworkers demonstrated that ONC inhibits growth and exerts cytotoxic effects against both drug-sensitive and chemo-resistant neuroblastoma cells [118]. However, the ONC-elicited cell death occurred without activation of caspases or cytochrome c release from mitochondria, suggesting that ONC induced cell death through a caspase-independent mechanism, probably by activating autophagy [118]. Conversely, Ardelt et al. discovered that ONC elicits apoptosis in some tumor cell lines through typical mechanisms foreseeing the activation of caspases. The destabilization of mitochondrial transmembrane potential also emerged by decreasing the ATP level and by affecting the expression of both B-cell lymphoma 2 (Bcl2) and the apoptosis regulator BAX (Bax) [80].

ONC also strongly inhibits the pancreatic adenocarcinoma cell proliferation through a mechanism involving Beclin1-mediated autophagic cell death. Additionally, it can sensitize pancreatic cancer cells to the standard chemotherapeutic agent gemcitabine, hence inhibiting cell growth and increasing apoptosis as well [108].

Since malignant mesothelioma and lung cancer are the most ONC-responsive tumors, several studies analyzed the ONC suppressive role against oncogenes involved in the pathogenesis of these malignancies. Fos-related antigen 1 (Fra1) is a transcription factor induced by lung carcinogens, such as cigarette smoke, and is a predominant component of the activator protein-1 transcription complex in asbestos-induced mesothelioma cells [114]. Additionally, the overexpression of a dominant negative Fra1 mutant inhibits the growth of these cells in soft agar [114,151]. Moreover, a high survivin expression is positively correlated with a more aggressive tumor cell phenotype, therefore shortening the animal survival by decreasing the response to chemotherapeutics [114]. Notably, by using a combination of ONC and rosiglitazone in mesothelioma cells, Ramos-Nino et al. registered either a reduction in proliferation rate or an apoptosis increase in concert with a decreased expression of Fra1 and survivin [114,151].

To identify all resulting pathways altered upon ONC exposure, the transcriptional profile of mesothelioma cell lines has been revealed with microarrays. By a transcriptomic analysis, Altomare et al. identified a subset of genes whose expression is modulated by ONC [146]. Many ONC-regulated genes resulted to be associated with cellular signal transduction, proliferation and differentiation, including genes previously found to be involved in mitogen-activated kinases signaling, cytokine-receptor interactions, Jak-STAT pathway and interleukins [152]. Furthermore, the activating transcription factor 3 (ATF3), which can suppress Ras-stimulated tumorigenesis, was upregulated by ONC [146,153]. Later, Vert et al. confirmed the overexpression of ATF3 induced by ONC in ovarian cell lines [103]. Altogether, these results offer a broad picture of gene activity and help to better comprehend the overall scenario related to mesothelioma cell response to the ONC therapeutic agent.

Recently, we registered, in the two human melanoma A375 and MeWo cell lines, that ONC decreases the expression level of the antiapoptotic protein Bcl2 and of the total content of the signal transducer and activator of transcription (STAT)3, together with its active forms and its upstream kinase Src [137]. Notably, STAT3 silencing strongly inhibits the tumor growth in a mouse melanoma model [154]. Hence, the ONC-related downregulation of both STAT3 and Bcl2 can partially explain either the apoptosis induction or the reduction in colony formation found in soft agar with these two melanoma cell lines [137].

Finally, an ONC pleiotropic effect affecting key intracellular proteins and counteracting their phenotype has been certified again in the highly malignant A375 cells [147]. ONC reduced the expression of cell cycle-regulated proteins, such as cyclins D1 and A2, and the activation of cyclin-dependent kinase-2 or retinoblastoma protein. In addition, it hindered the survival signaling pathways affecting ERK, protein kinase B (Akt) and the cAMP response element-binding protein (CREB) [147]. Then, ONC lowered the protein expression of oncogenes through tyrosine kinase activity receptors, such as the hepatocyte growth factor receptor (c-Met) and the tyrosine-protein kinase receptor UFO (AXL). Moreover, it slowed down the expression of both transcription factor Fra1 and HIF1α, i.e., the hypoxia-inducing factor 1α [147]. Finally, sirtuin-1, zonula occludens-1, urokinase plasminogen activator surface receptor (uPAR) and SRY (sex determining region Y)-box 2 (SOX2) were also downregulated by ONC in A375 melanoma cells [147], in line with the low invasiveness and low metastatic potential effects previously found [109].

In conclusion, the broad spectrum of ONC effects reported by many studies was confirmed with cell lines deriving from different tumor types, hence presupposing assorted targets existing in distinct contexts. In any case, ONC acts simultaneously on multi-intracellular targets, suggesting it may affect many RNA species involved in the regulation of gene and protein expression, as is discussed hereafter.

### 4.6. ONC Antiviral Activity

RNases play a key role in immune defense, since it is well known their engagement in the protection of cells and organisms from microbes, especially viruses. Interestingly, Boix and coworkers recently found that RNase2 exerts its antiviral activity, in a cell line of human macrophages, by cleaving tRNAs and other ncRNA species [60,155].

The first hurdle against viral infection passes through the induction of type I interferons, which activate RNase L and interferon-stimulated gene-20, both displaying ribonucleolytic activity specifically devoted to viral RNAs [156]. Thereby, RNases are now considered the basis for designing new antiviral preparations [81]. As it regards ONC, the antiviral effects nowadays registered are summarized in Table 2.

First, after its discovery, Youle et al. found that ONC inhibits HIV-1 infection in H9 leukemia cells at concentrations that did not block the protein synthesis of uninfected H9 cells [157]. The antiviral activity was then investigated by Saxena et al. in both H9 and U937 cells: indeed, ONC directly inhibited HIV-1 infection within viable and dividing cells by inducing a large decrease in all HIV-1 RNA transcript levels [158]. The most dramatic decrease in HIV-1 RNA levels lasted up to four days, just when the ONC antiviral effect began to decrease, although the antiviral effects were renewed upon repeated ONC applications [158]. Importantly, ONC inhibits viral production even when it is administered after HIV1 cells infection. Hence, the authors concluded that ONC could become a promising therapeutic agent against HIV-1 infection, since its dosage and effectiveness well coincide with ONC safe regimens utilized in clinical trials against cancer [158]. Similarly to antitumor activity, the ONC antiviral potential was correlated with its ribonucleolytic activity even if the molecular mechanism was unknown at that time. Therefore, the same authors mechanistically investigated ONC antiviral effects occurring in HIV-1 infection [159]: HIV-1 replication requires the enzymatic activity of reverse transcriptase (RT) that in turn requires a primer, which for HIV-1 is cellular tRNA^Lys3^, to initiate the DNA synthesis. Considering that ONC, as is discussed later, can selectively degrade tRNAs, the authors analyzed if RT priming could be inhibited by ONC, finding degradation products of tRNA^Lys3^ in HIV-1 infected H9 cells subsequently treated with sublethal ONC concentrations [159]. Notably, Suhasini and Sirdeshmukh also measured the ONC cleavage specificity present in the in vitro-transcribed tRNA^Lys3^ [160].

**Table 2:** Effects of ONC on viruses.

Virus	Cell Type	General Biological Effect(s)	Measured Effects	Reference
HIV-1 (leukemia cells)	H9	Syncytial cell aggregate ↓ Viral replication ↓ No cytotoxicity	HIV-1 p24 antigen ↓	Youle, R.J. [157]
HIV-1 (leukemia cells)	H9 and U937	Viral replication ↓ No cytotoxicity	HIV-1 p24 antigen ↓ HIV-1 RNA degradation ↑	Saxena, S.K. [158]
HIV-1 (leukemia cells)	H9	No cytotoxicity No difference in total tRNAs	Specific degradation of tRNA^Lys1,2^ tRNA^Lys3^_,_ tRNA^Phe^	Saxena, S.K. [159]
(HIV-1)	Synthetic t-RNA^Lys3^	Cleavage at the variable loop	tRNA^Lys3^ degradation	Suhasini, A.N. [160]
HIV-1	HIV-infected colorectal explants LPS stimulation	HIV infection ↓ Inflammation ↓	HIV-1 p24 antigen ↓ Cytokines, chemokines and inflammatory markers in the supernatant ↓ (dose-dependently)	Brand, R.M. [161]
Ebola (EBOV) Mouse-adapted EBOV	In vitro: Vero cells Vero E6 cells In vivo in mice	Cell viral infection ↓ Sera, kidneys, liver and spleen in vivo viral infection (pre- and postexposure) ↓	Viral load determination in sera, kidneys, liver and spleen ↓ Animal survival ↑	Hodge, T. [162]
Human Papilloma HPV-11	A431 (epidermoid carcinoma) Phase I Clinical Trial 42 patients. In vivo topic application	Cell viral infection ↓ Topic viral infection ↓	Viral transcript ↓ Clinical efficacy ↑ (83% of patients clinical healing; 17% reached 50% symptom reduction)	Squiquera, L. [163]
Rabies (RABV)	Cell types: baby hamster kidney mouse neuroblastoma bat primary fibroblast In vivo: Syrian Hamster	Cell-to-cell infection ↓ No results in animals	Animal survival = RABV release (dose dependently) ↓	Smith, T.G. [164]

Since the gastrointestinal tract is a major site for initiating HIV-1 infection, the ex vivo colorectal challenge model is often used to determine the effectiveness of drugs devoted to HIV-1 prevention. Brand et al. cultured colorectal biopsies for two weeks with a large range of ONC concentrations without observing any cytotoxicity sign [161]. Indeed, ONC reduced the inflammatory environment that might facilitate HIV-1 infection, decreased the HIV-1-elicited expression of many proinflammatory cytokines and upregulated the transcription factor ATF3 [103,146,153], which in turn prevented viral genome replication [161]. Thereafter, a valuable strategy was designed by Callis et al. to produce ONC variants that can be recognized and cleaved by HIV-1 proteases [165]. Uncleaved ONC variants displaying no cytotoxicity can certainly enter human T-lymphocytes, but they can be cleaved by viral proteases and become cytotoxic when embedded in HIV-1-infected cells [165]. Undoubtedly, the creation of ONC-based zymogens would provide a versatile option to manage ONC enzymatic activity, therefore targeting its toxicity only to cells living in a specific disease state [165].

Importantly, the antiviral effect of ONC is not limited to HIV infection but emerged against other viruses as well, such as Ebola, human papilloma, or Rabies viruses, and more recently also against SARS-CoV-2.

Ebola viruses cause a severe form of viral hemorrhagic fever, they are endemic in central Africa, and, unfortunately, no prophylaxis or successful treatment are available today [166]. In addition, the persistence for several months of Ebola virus in body fluids raises the possibilities of recurrence and reinfection [166]. More recently, Hodge et al. demonstrated that ONC reduces titers of this virus in infected cells. Additionally, it protects mice when administered either pre or post viral exposure [162]. It is likely that ONC directly targets viral dsRNA intermediates to break the viral life cycle. Thus, ONC could become a promising agent for further development of anti-Ebola virus therapy [162].

Human papillomaviruses (HPV), the causative agents of anus–genital warts, are the most prevalent sexually transmitted infectious agents for the eradication of which no specific antiviral therapy is still available [167]. Squiquera et al. reported an ONC-specific activity against HPV-11 and low toxicity in A431 cells cultures [163]. Then, they used ONC as a topical application to treat clinical HPV-11 infections in a Phase I study enrolling 42 participants. Indeed, 1 mg/mL/week of topical ONC solution was moderately well-tolerated and caused a significant reduction in the clinical severity score. These in vivo results are promising and suggest extending the studies also toward more malignant HPV variants, such as HPV-16 and -18.

The rabies virus (RABV) is transmitted to the host by saliva through the bite of an infected animal. Currently, no approved RABV-specific antiviral drug is available, while Smith et al. investigated the ONC antiviral activity against RABV either in vitro or in vivo [164]. Unfortunately, although RABV release was inhibited in a dose-dependent manner in neuronal, epithelial and primary fibroblast cells, the effect of ONC administration at 0.1 mg/kg was not significantly different for both clinical onset, or death, in a hamster animal model [164]. Hence, additional studies are required to determine if an effective dose, the delivery route, or new drug formulations could prevent rabies in animal models [164]. Indeed, since the in vivo effectiveness against RABV likely requires ONC crossing the brain–blood barrier, novel delivery methods should be designed to overcome this hurdle.

Coronaviruses (CoVs) were first identified at the beginning of 1900 in domestic animals, and their importance rose since 2003 when three severe CoV human diseases emerged: the Severe Acute Respiratory Syndrome (SARS), the Middle East Respiratory Syndrome (MERS) and the SARS-CoV-2 Infection Disease-19 (COVID-19) [168,169,170]. COVID-19 symptoms are similar to SARS, including fever, pneumonia, cough, and in severe cases, serious dyspnea and lung infiltration [171]. The high infectivity of SARS-CoV-2, the pathogen responsible for COVID-19, caused a rapid spread worldwide and over five million deaths in two years after the disease onset. Despite the fast vaccine development achievement [172], as well as the recent approvals of virus-directed therapeutics, this pandemic is far from reaching eradication [171]. In June 2020, the Orgenesis and Leidos Company started a clinical trial with ONC in COVID-19 infections, after U.S. FDA marketing approval (https://adisinsight.springer.com/drugs/800001659. accessed on: 17 May 2022). ONC was utilized as an agent with antiviral broad-spectrum that catalyzed RNA degradation [173]. However, no update is available so far about this experimentation.

It is noteworthy that, among the strategies combatting SARS-CoV-2, the nucleic acid-based therapeutics, including the use of antisense oligonucleotides, miRNAs, small interfering RNAs and others, could be promising [174]. In this regard and despite its direct antiviral activity, ONC could also counteract the infection disease modulating the intracellular interfering RNA species. The mechanism of ONC antiviral activity is linked to its ribonucleolytic activity that is discussed in the next section and can include direct virus inhibition and/or alterations in the host cell gene expression [175].

### 4.7. Preferential targets of ONC Ribonucleolytic Activity: tRNAs and miRNAs’ precursors

Ardelt et al. firstly reported that ONC cytostatic/cytotoxic activity against A253 squamous carcinoma cells is paralleled by its enzymatic specificity [71]. In fact, ONC alkylation of the catalytic His residues with iodoacetic deleted the antitumor effect of the enzyme [71], as subsequently confirmed in other cell systems [176].

As reported by Saxena et al., intracellular dsRNAs are ONC substrates, thus generating differently sized dsRNA fragments which could trigger a cellular response by utilizing multiple signaling pathways [177]. Indeed, unlike RNase A, ONC concentrations ranging from 10^−8^ to 10^−6^ M cleave an in vitro-transcribed GAPDH-dsRNA substrate by generating a cocktail of 20–400 bp dsRNA fragments [177]. Hence, the ability of ONC to digest dsRNAs fits well with its antiviral activity [158,159].

Upon measuring [^14^C]-Leu incorporation and registering an IC50% of 10^−7^ M, Wu et al. reported that ONC is cytotoxic against 9L glioma cells by inhibiting protein synthesis [84]. An ONC concentration-dependent capability to digest tRNAs as well as 28S and 18S ribosomal RNA (rRNA), but not 5.8S and 5S rRNAs, was found [84]. Afterwards, other authors proved that ONC causes a potent protein synthesis inhibition through the inactivation of cellular tRNAs: indeed, ONC can degrade them in a reticulocyte lysate even when it is administered at a 1000-fold lower concentration than that required to digest rRNA species. Moreover, the re-addition of tRNAs to ONC-treated lysates can restore protein synthesis [178].

Saxena et al. investigated the ONC target specificity in intact H9 leukemia cells, finding it is cytotoxic above 10^−7^ M [159]. From this concentration to 10^−5^ M, ONC digests tRNA but neither rRNA nor mRNA species in cells [159]. Conversely, no selectivity for different RNAs was registered by directly adding ONC to a mixture of all RNA species previously purified from the reticulocyte lysate. This suggests the protein-RNA complex could protect both intracellular mRNAs and rRNAs against ONC ribonucleolytic activity in physiological conditions [159]. The same authors noted the presence of 30–40 residues long products deriving from tRNA degradation in cells, thus indicating that ONC can cleave tRNAs at different sites and with sequence specificity. Unexpectedly, ONC induced new tRNA synthesis in cells, so that the total tRNA turnover was enhanced [159].

Again, Abraham et al. compared the ONC cytotoxic mechanism with those of bleomycin antibiotics in SF539 glioma cells [179], showing that both agents can inhibit protein synthesis by digesting tRNAs, in parallel with the increase in cell cytotoxicity. However, none of the major bleomycin tRNA cleavage sites corresponded to the ONC ones [179]. Ardelt et al. claimed that ONC cytotoxicity does not fit with a nonspecific inhibition of protein synthesis alone [180]. Indeed, ONC induced a cell cycle arrest in G1 phase occurring later than ONC administration. Moreover, the arrest correlates in some cases with an increase in the expression levels of the cyclin-dependent kinases inhibitors, instead of a generalized downregulation of protein expression [139]. Finally, the ONC-elicited apoptosis seemed to be different from that induced by the classic protein synthesis inhibitors. Moreover, the kinetics of cell response to ONC required a 24–48 h delay, this effect being not surprising considering it should involve an altered gene expression profile rather than the abrupt global suppression of protein synthesis [180].

As discussed previously, a major part of the whole genomic transcriptional output are noncoding RNAs, a large fraction of which are involved in the regulation of protein expression. Hence, we agree with Ardelt et al. who asserted that miRNAs could assume a pivotal role upon ONC action by operating as small interfering RNAs. In addition, they hypothesized that cleaved tRNA products could act as RNA interfering in silencing particular genes. From this, ONC may act like a Dicer enzyme to generate small RNA species able to modulate cellular translational processes [180].

By comparing RNase A and ONC effects on rRNAs or tRNAs, Suhasini and Sirdeshmukh discovered the basis of ONC-specific cleavages, although they registered that both RNAs can be targeted and digested by both enzymes [181]. They found that rRNAs are the best RNase A targets, while tRNAs are the best for ONC: indeed, tRNA degradation was detectable upon administering 50 nM ONC, whereas rRNA digestion occurred only with 4–8 µM ONC [181]. ONC and RNase A activities were also compared by targeting a specific purified tRNA substrate. While RNase A cleavage resulted in many different sized fragments, the ONC-elicited tRNA digestion was more specific, generating a major product plus few other fragments [181]. Moreover, no modification of the cleavage pattern was observed over increasing the delay from ONC administration. In addition, the proportion of fragments deriving from the cleavage major site needed the conservation of a tRNA secondary structure because ONC produced the same fragments but with no preferential cleavage sites upon digesting a denatured substrate [181]. Again, Suhasini and Sirdeshmukh examined the sequence specificity of ONC cleavage sites in tRNA^Phe^, tRNA^Lys^ and tRNA^fMet^, reporting the cut occurs at the G–G bond, especially if these two nucleotides belong to the UGG sequence. In any case, the preferential ONC cleavage sites were located in the tRNA variable loop or in its D-arm [181]. This specificity may be related to ONC biological functions, as well as to RNA interfering processes. Thereafter, the same authors investigated the mechanism of ONC antiviral activity for which tRNAs can be important targets by acting as primers for viral replication [160]. In particular, as previously mentioned, cellular tRNA^Lys3^ is the primer for HIV-1 reverse transcriptase [159]. ONC inhibits HIV-1 replication by cutting the G–G bond (corresponding to G44-G45 or G45-G46) in the variable loop of a GGG tRNA^Lys3^ triplet [160]. Then, three different tRNA^Lys3^ mutated sequences introduced in the loop cleavage site did not impair tRNA^Lys3^ cleavage, since in this case ONC can be active vs. nearby-located sites regardless of the sequence context [160]_,_ This suggests a possible contribution of the tRNA^Lys3^ secondary structure in the process, as confirmed by the data reported by Lee and Raines [82].

Again, other ncRNA species can mediate ONC cytotoxicity. In malignant pleural mesothelioma cells, Goparaju et al. demonstrated that ONC affects cell proliferation, invasion and apoptosis by inducing several alterations in miRNAs expression profiles. The most significant were an upregulation of miR-17* and a downregulation of miR-30c [143]. Remarkably, upon transfecting cells, respectively, with miR-17* mimic or with miR-30c inhibitor, cell proliferation, invasion, migration and soft agar colony formation were affected comparably to what was achieved with ONC [143]. Likewise, the expression level of the mentioned miRNAs targets, such as gene and protein NF-κB, as well as ATP-binding cassette subfamily B member 1 (ABCB1), was decreased either upon ONC incubation or upon transfecting miR-17* mimic or a miR-30c inhibitor [143]. Hence, the authors suggested that ONC exerts its antitumor effect likely by modulating the expression of such miRNAs.

Interestingly, Truini et al. suggested valuable therapeutic implications of either miRNA mimics or of ONC in malignant pleural mesothelioma, proposing them as new agents to better counteract this aggressive disease by modulating the expression of specific miRNAs [182].

Qiao et al. investigated instead the molecular mechanism by which ONC affects miRNAs expression. Initially, finding an ONC-elicited downregulation of both miR-155 and miR-21 in the Msto-211h mesothelioma cell line, they measured the ONC activity on chemically synthesized 23-nt matures and 65-nt precursors of such miRNAs; whereas the precursor strand was significantly cleaved by ONC, the mature miRNA did not, suggesting the precursors are preferential ONC targets [183]. The authors also discovered that ONC predominantly cleaves these precursors at the UG or UU nucleotides, speculating that miRNA precursors can be cleaved by ONC because they are similar to tRNAs [116]. This can occur either because of their length or because they display hairpins and secondary structures.

Recently, we analyzed the expression level of several onco-suppressor miRNAs and their targets in two BRAF-mutated melanoma cell lines [147]. The most upregulated miRNAs in ONC-treated A375 and FO-1 cell lines resulted to be miR-20a-3p and miR-34a-5p. Their upregulation correlated well with the cell proliferation arrest and with the decrease in cell migration, invasion and soft agar colony formation found in ONC-treated A375 cells [109]. Remarkably, predicted targets of miR-20a-3p and miR-34a-5p are many mRNAs codifying for proteins that are downregulated in ONC-treated melanoma cells (Table 1). Indeed, some cyclins and cyclin-dependent kinases controlling the G1/S checkpoints of the cell cycle are poorly expressed in ONC-treated A375 cells, in parallel with the overexpression of miR-20a-3p and miR-34a-5p. Likewise, several kinases and transcription factors involved in prosurvival signaling pathways were downregulated [137,147]. Other proteins, whose expression was decreased by ONC, are instead involved in cell migration, invasion and prometastatic potential increase. Finally, data obtained from transfecting miR-20a-3p or miR-34a-5p inhibitors in the presence of ONC showed a reversion of ONC-elicited downregulation of cyclin A2, c-Met, AXL and Fra1, which are targets of such miRNAs. This suggests the ONC antitumor effect in A375 melanoma cells may be mediated by these overexpressed miRNAs [147].

It is noteworthy that lncRNAs also play an important function either in cancer or in viral infections. Recently, Lu et al. [59] demonstrated that the lncRNA MALAT1 (metastasis-associated lung adenocarcinoma transcript-1) can be processed by RNase P and RNase Z. In this way, these RNases, that are also involved in tRNA biogenesis, generate a small ncRNA called mascRNA [59]. By mimicking tRNA structures, mascRNA can regulate virus translation in plants [184] and promote cell proliferation in eukaryotes [59]. The authors conclude that mascRNA could be part of the tumor-promoting mechanism of MALAT1. In addition, and remarkably, investigations on mascRNA could help to unveil the functions of tRNA-like structures in mammalian cells [59]. In this regard, the ability of ONC in processing tRNAs could also be directed to tRNA-like ncRNAs managing.

## 5. Conclusions

It now seems clear that the pleiotropic effects of ONC derive from its ability to generate many different ncRNAs through a ribonucleolytic cleavage occurring not randomly, since ONC preferentially recognizes, at least in pre-miRNA and tRNA substrates, a specific sequence and/or a peculiar secondary/tertiary structure.

ONC cleaves tRNAs at their D-, TψC- and anticodon-loops, which are the same target sites of Dicer or ANG RNases. Hence, ONC could be considered as another RNase able to produce both tiRNAs and tRFs.

ONC effects are cell-type specific and depend to the pathological state of cells because its pre-miRNAs and tRNAs substrates are otherwise expressed either in normal or in pathogenic conditions. Therefore, ONC can affect protein expression by generating RNA fragments displaying interfering properties, particularly in pathological conditions. This, therefore, results in a reversion of the malignant phenotype.

Finally, very promising is the ONC ability to hinder virus spread by interfering with the availability of the primers necessary for viral replication. Importantly, this effect is not paralleled by damaging the host replication machinery. Therefore, the possibility to trigger ONC activity directly by the virus only in infected cells is intriguing [165].

Many queries, however, remain to be answered:

1. Could lncRNA, circRNA or other ncRNA species be substrates for ONC activity, such as miRNAs and tRNAs are?
2. What is the intracellular activity of each tsRNAs generated by ONC?
3. Could ONC counteract other virus species? To this end, it is worth mentioning the multiple findings assessing the pleiotropic effects exerted by ONC, as well as the tests now devoted to measure its activity on SARS-CoV-2.

From what was described, we can confirm that ONC might become therapeutically efficacious since it often induces either potentiation and/or antiresistance effects if it is administered together with drugs considered as gold standard for specific tumor cells. Instead, for future applications, a promising strategy could be related to the identification and sequencing of the main products derived by ONC-elicited tRNA cleavage in pathological conditions. Indeed, this might become a tool for delivering such synthetic, “therapeutic” tsRNAs to specifically operate into malignant cells.

## Data Availability

Not applicable.
